# Supplementary material for: A RAS-Independent Biomarker Panel to Reliably Predict Response to MEK Inhibition in Colorectal Cancer
Source: Cancers (Basel). 2022 Jul 1;14(13):3252. doi: 10.3390/cancers14133252 (PMC9265111; doi:10.3390/cancers14133252)
Supplement: Supplementary file 1 [file cancers-14-03252-s001.zip › Supplementary Files/Figure S4.pdf]

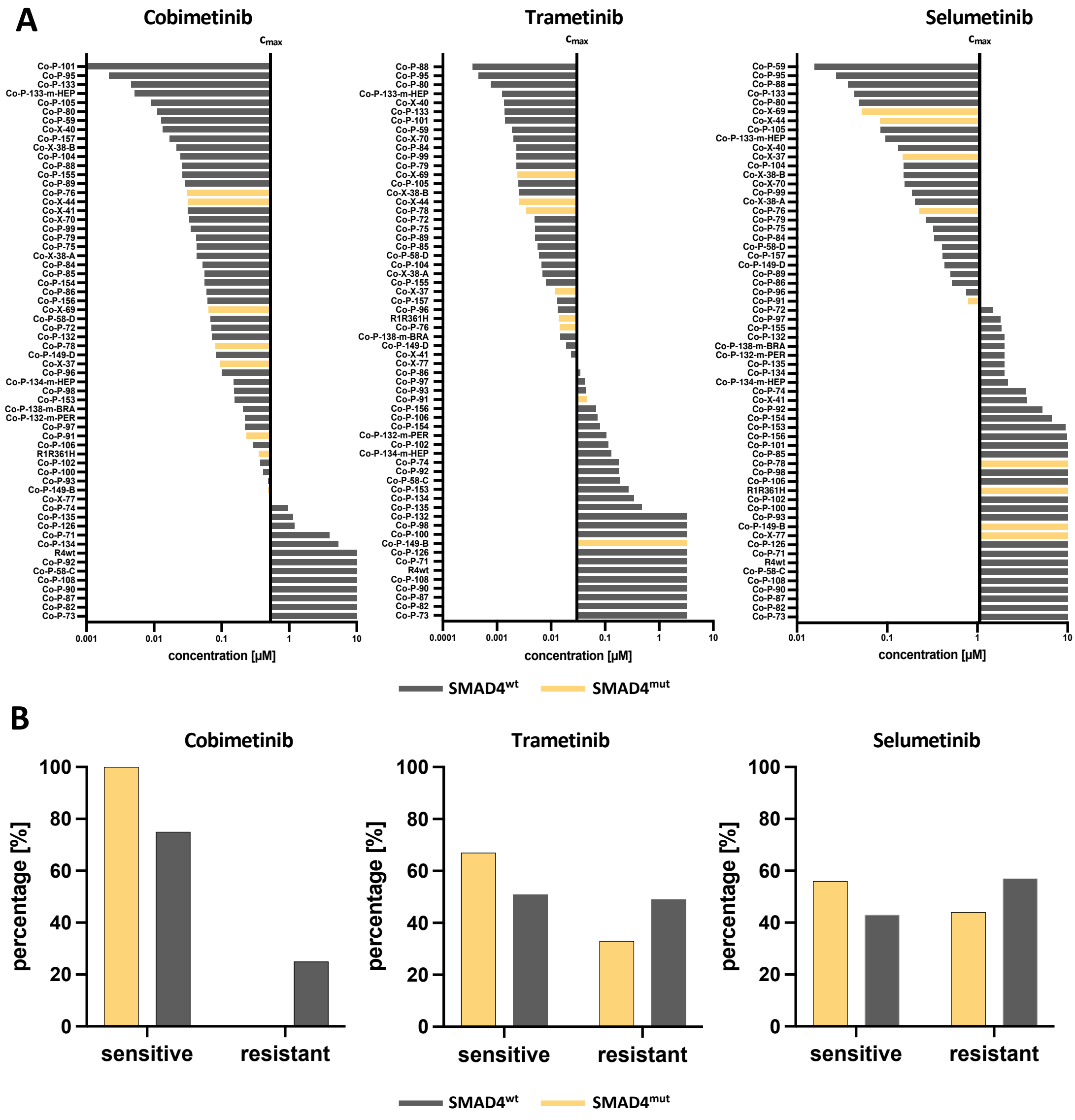

n = 62	Cobimetinib		Trametinib		Selumetinib	
**Effect size**	**Value**	**95 % CI**	**Value**	**95 % CI**	**Value**	**95 % CI**
**Sensitivity**	0.184	0.099 to 0.314	0.182	0.0861 to 0.344	0.179	0.0788 to 0.356
**Specificity**	1.000	0.772 to 1.000	0.897	0.736 to 0.964	0.882	0.734 to 0.953
**Positive Predictive Value**	1.000	0.701 to 1.000	0.667	0.354 to 0.879	0.556	0.267 to 0.811
**Negative Predictive Value**	0.245	0.149 to 0.376	0.491	0.361 to 0.621	0.566	0.433 to 0.691
	p 0.184		p 0.483		p 0.719	
