# Supplementary material for: A RAS-Independent Biomarker Panel to Reliably Predict Response to MEK Inhibition in Colorectal Cancer
Source: Cancers (Basel). 2022 Jul 1;14(13):3252. doi: 10.3390/cancers14133252 (PMC9265111; doi:10.3390/cancers14133252)
Supplement: Supplementary file 1 [file cancers-14-03252-s001.zip › Supplementary Files/Figure S5.pdf]

A

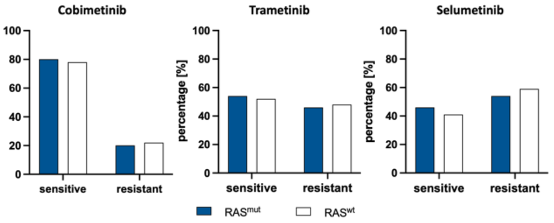

n = 62	Cobimetinib		Trametinib		Selumetinib	
**Effect size**	**Value**	**95 % CI**	**Value**	**95 % CI**	**Value**	**95 % CI**
**Sensitivity**	0.571	0.433 to 0.700	0.576	0.408 to 0.728	0.460	0.366 to 0.557
**Specificity**	0.462	0.232 to 0.709	0.448	0.284 to 0.625	0.590	0.492 to 0.681
**Positive Predictive Value**	0.800	0.641 to 0.900	0.543	0.382 to 0.695	0.529	0.425 to 0.630
**Negative Predictive Value**	0.222	0.106 to 0.408	0.482	0.307 to 0.660	0.522	0.431 to 0.612
	p >0.999		p >0.999		p 0.568	

D

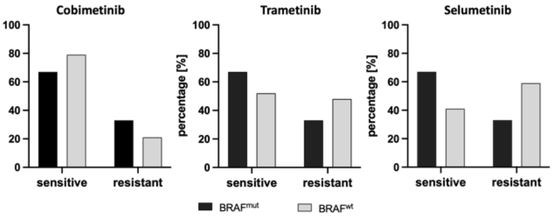

n = 62	Cobimetinib		Trametinib		Selumetinib	
**Effect size**	**Value**	**95 % CI**	**Value**	**95 % CI**	**Value**	**95 % CI**
**Sensitivity**	0.083	0.033 to 0.196	0.121	0.048 to 0.273	0.148	0.059 to 0.325
**Specificity**	0.857	0.601 to 0.975	0.931	0.780 to 0.988	0.943	0.814 to 0.990
**Positive Predictive Value**	0.667	0.300 to 0.941	0.667	0.300 to 0.941	0.667	0.300 to 0.941
**Negative Predictive Value**	0.214	0.127 to 0.338	0.482	0.357 to 0.610	0.589	0.459 to 0.708
	p 0.610		p 0.676		p 0.390	

B

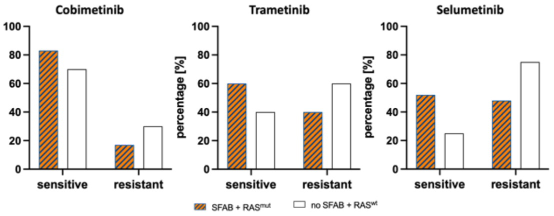

n = 62	Cobimetinib		Trametinib		Selumetinib	
**Effect size**	**Value**	**95 % CI**	**Value**	**95 % CI**	**Value**	**95 % CI**
**Sensitivity**	0.714	0.576 to 0.822	0.758	0.590 to 0.872	0.815	0.633 to 0.918
**Specificity**	0.462	0.232 to 0.709	0.414	0.255 to 0.593	0.429	0.280 to 0.591
**Positive Predictive Value**	0.833	0.694 to 0.917	0.595	0.445 to 0.730	0.524	0.377 to 0.666
**Negative Predictive Value**	0.300	0.146 to 0.519	0.600	0.387 to 0.781	0.750	0.531 to 0.888
	p 0.319		p 0.181		p 0.057	

E

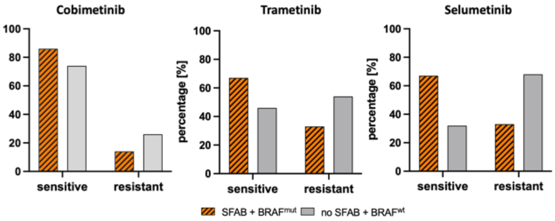

n = 62	Cobimetinib		Trametinib		Selumetinib	
**Effect size**	**Value**	**95 % CI**	**Value**	**95 % CI**	**Value**	**95 % CI**
**Sensitivity**	0.360	0.241 to 0.499	0.424	0.272 to 0.592	0.519	0.340 to 0.693
**Specificity**	0.786	0.524 to 0.924	0.759	0.579 to 0.878	0.800	0.641 to 0.900
**Positive Predictive Value**	0.857	0.654 to 0.950	0.667	0.454 to 0.828	0.667	0.454 to 0.828
**Negative Predictive Value**	0.256	0.149 to 0.402	0.537	0.388 to 0.679	0.683	0.530 to 0.804
	p 0.356		p 0.180		p* 0.014	

C

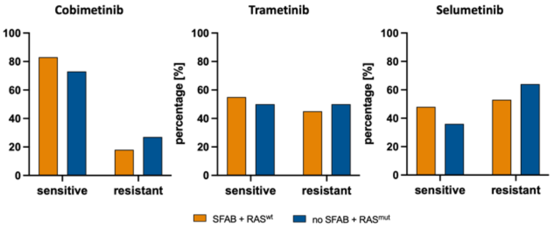

n = 62	Cobimetinib		Trametinib		Selumetinib	
**Effect size**	**Value**	**95 % CI**	**Value**	**95 % CI**	**Value**	**95 % CI**
**Sensitivity**	0.674	0.534 to 0.788	0.667	0.496 to 0.803	0.704	0.515 to 0.842
**Specificity**	0.462	0.232 to 0.709	0.379	0.227 to 0.560	0.400	0.256 to 0.564
**Positive Predictive Value**	0.825	0.681 to 0.913	0.550	0.398 to 0.693	0.475	0.329 to 0.625
**Negative Predictive Value**	0.273	0.132 to 0.482	0.500	0.307 to 0.693	0.636	0.430 to 0.8023
	p 0.516		p 0.793		p 0.435	
