# Supplementary material for: A RAS-Independent Biomarker Panel to Reliably Predict Response to MEK Inhibition in Colorectal Cancer
Source: Cancers (Basel). 2022 Jul 1;14(13):3252. doi: 10.3390/cancers14133252 (PMC9265111; doi:10.3390/cancers14133252)
Supplement: Supplementary file 1 [file cancers-14-03252-s001.zip › Supplementary Files/Figure S6.pdf]

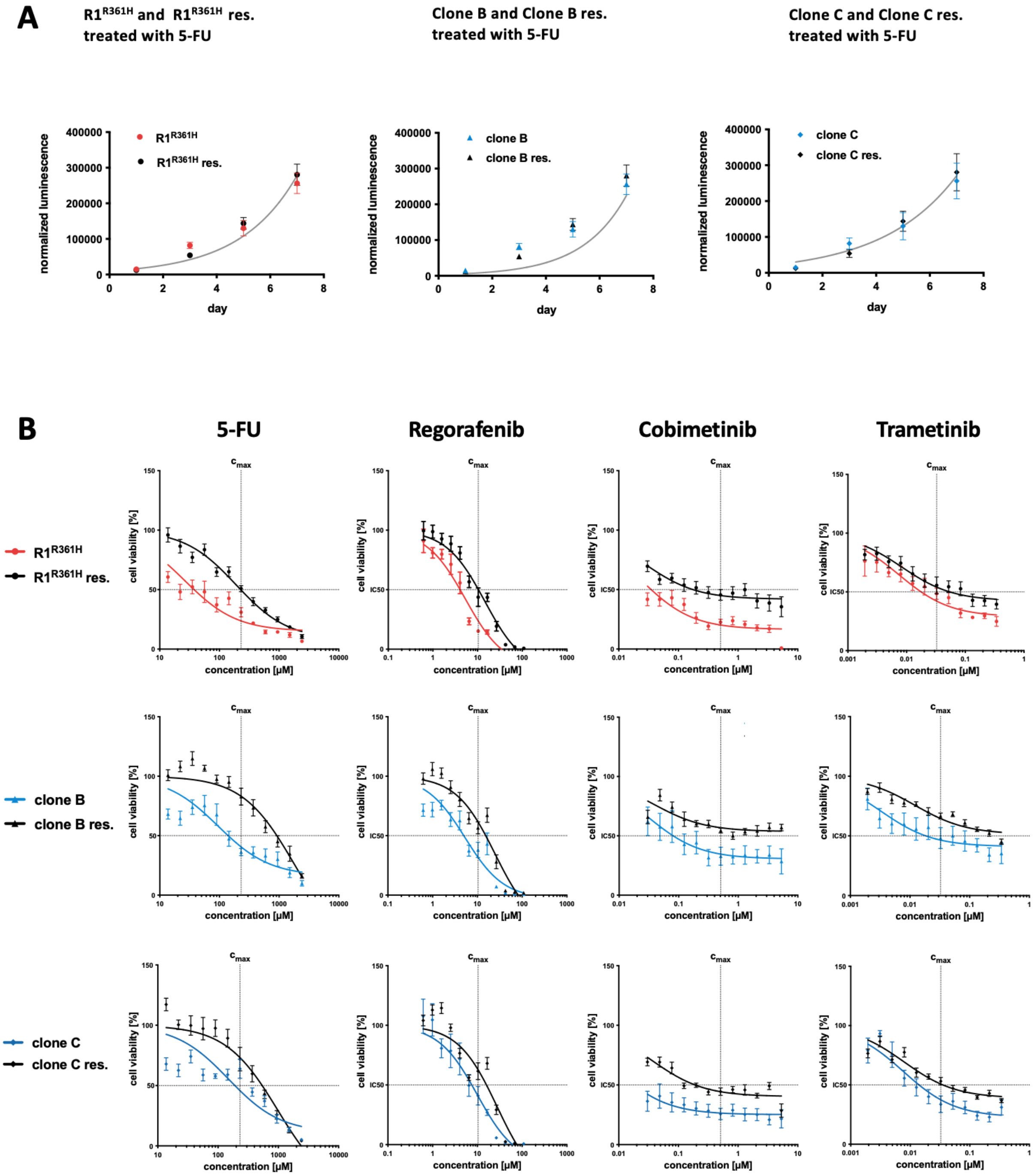
A
R1R361H and R1R361H res. treated with 5-FU
Clone B and Clone B res. treated with 5-FU
Clone C and Clone C res. treated with 5-FU
normalized luminescence
day
R1R361H
R1R361H res.
clone B
clone B res.
clone C
clone C res.
B
5-FU
Regorafenib
Cobimetinib
Trametinib
cmax
cell viability [%]
IC50
concentration [µM]
R1R361H
R1R361H res.
clone B
clone B res.
clone C
clone C res.
