# Supplementary figures and images for: A RAS-Independent Biomarker Panel to Reliably Predict Response to MEK Inhibition in Colorectal Cancer
Source: Cancers (Basel). 2022 Jul 1;14(13):3252. doi: 10.3390/cancers14133252 (PMC9265111; doi:10.3390/cancers14133252)

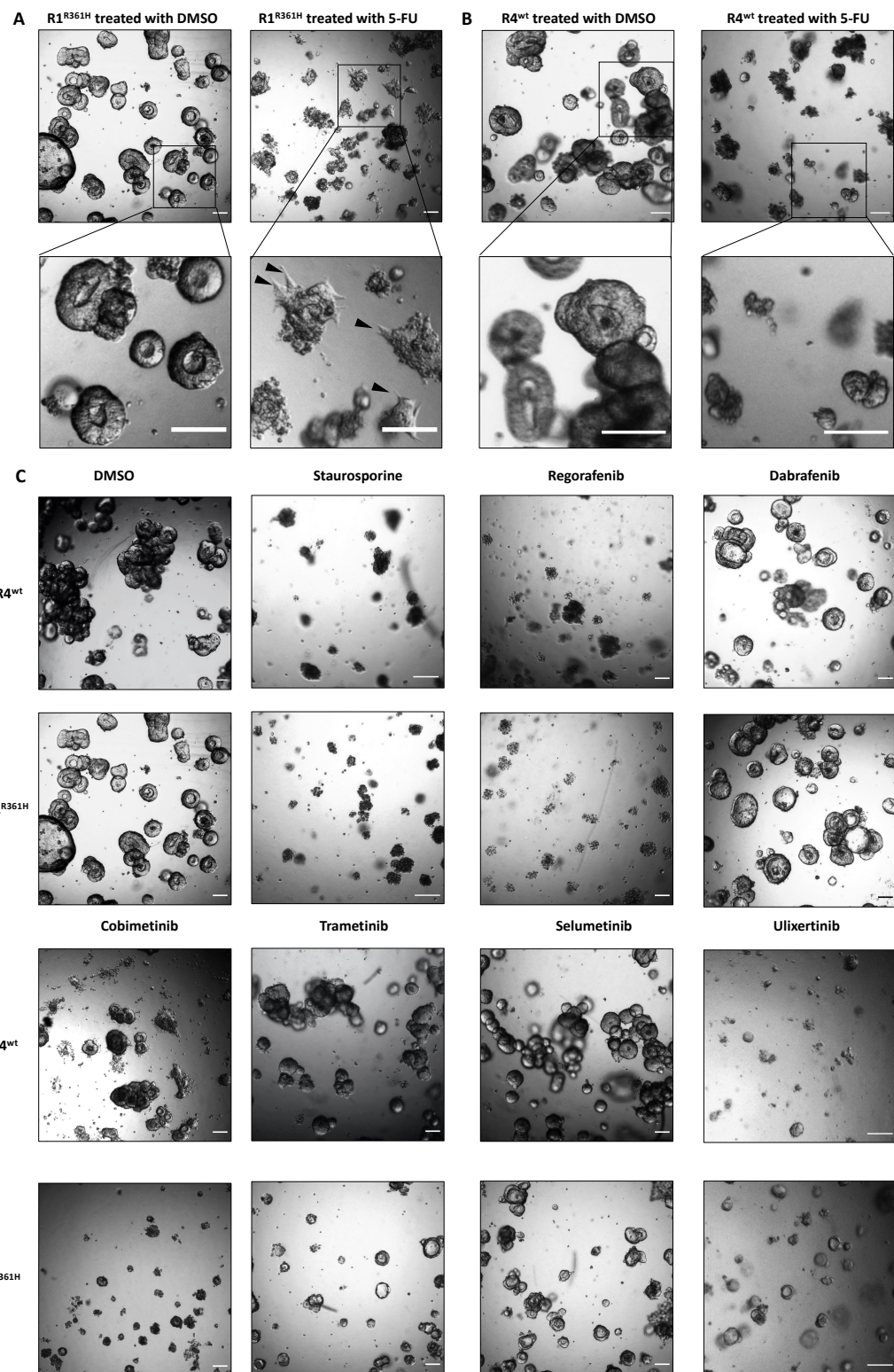

Supplement: Supplementary file 1 [file cancers-14-03252-s001.zip › Supplementary Files/Figure S1.pdf]

A

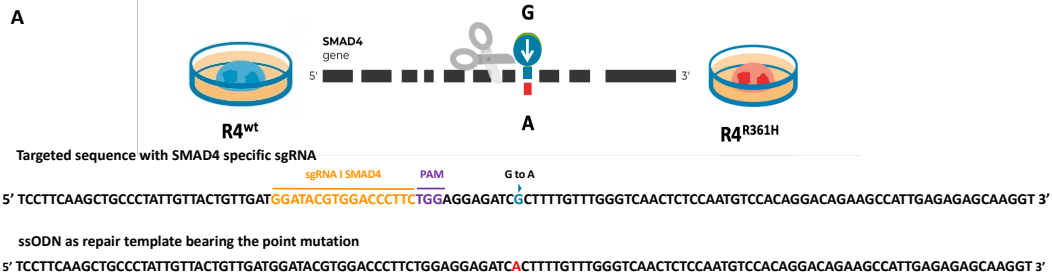

B

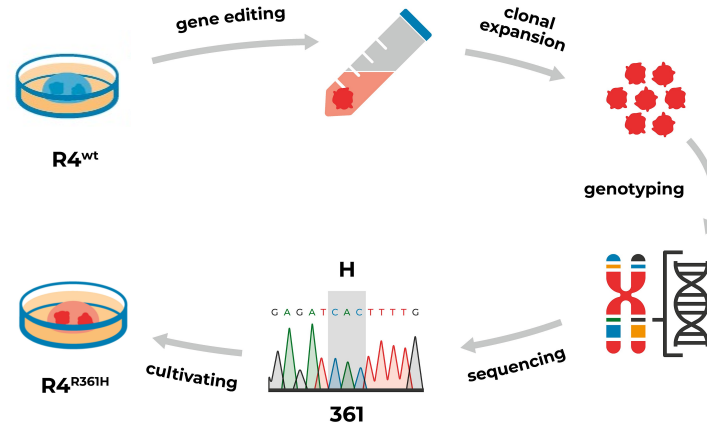

C

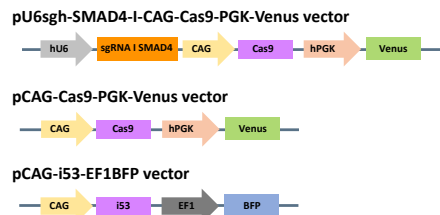

D

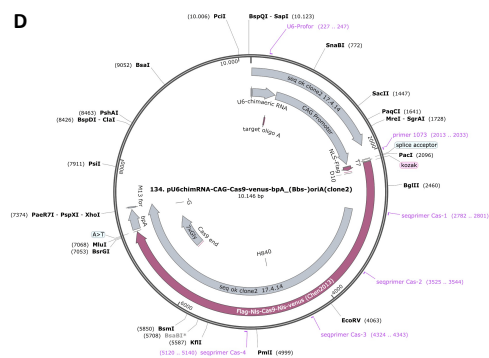

E

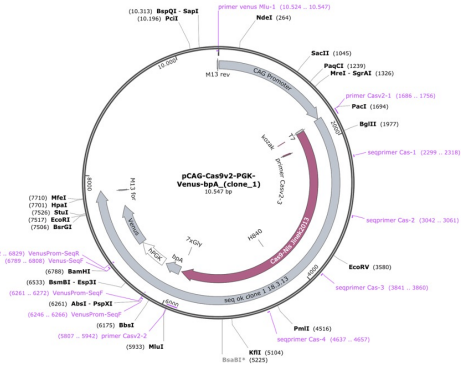

F

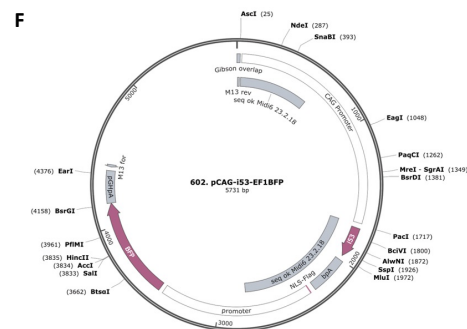

Supplement: Supplementary file 1 [file cancers-14-03252-s001.zip › Supplementary Files/Figure S2.pdf]
