# Supplementary material for: A RAS-Independent Biomarker Panel to Reliably Predict Response to MEK Inhibition in Colorectal Cancer
Source: Cancers (Basel). 2022 Jul 1;14(13):3252. doi: 10.3390/cancers14133252 (PMC9265111; doi:10.3390/cancers14133252)
Supplement: Supplementary file 1 [file Figure S3.pdf › Supplementary Files/Figure S3.pdf]

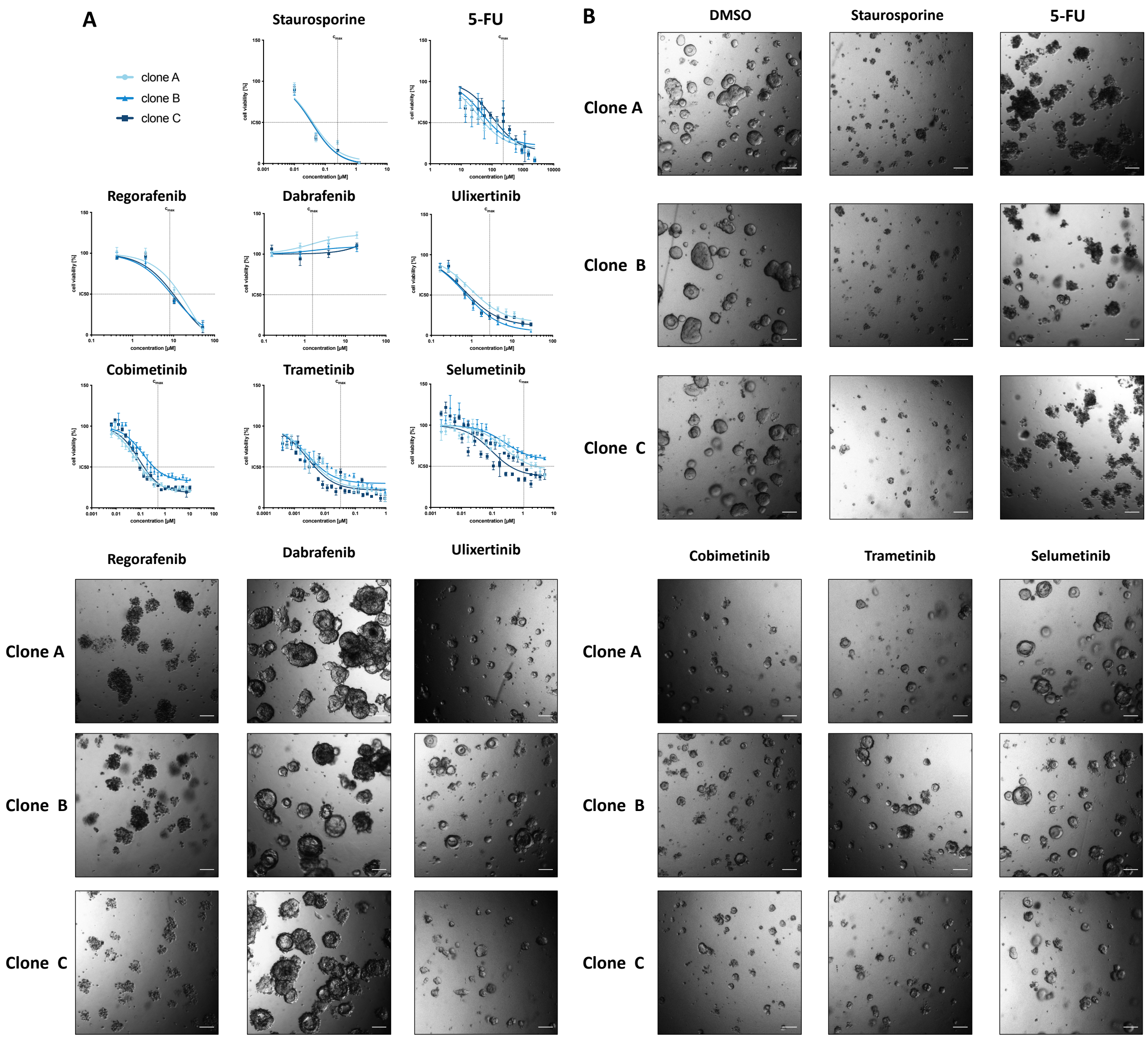
A
Staurosporine
5-FU
clone A
clone B
clone C
Regorafenib
Dabrafenib
Ulixertinib
Cobimetinib
Trametinib
Selumetinib
Regorafenib
Dabrafenib
Ulixertinib
Clone A
Clone B
Clone C
B
DMSO
Staurosporine
5-FU
Clone A
Clone B
Clone C
Cobimetinib
Trametinib
Selumetinib
Clone A
Clone B
Clone C
